# Transoral, retromolar, para-tonsillar approach to the styloid process in 6 patients with Eagle’s syndrome

**DOI:** 10.4317/medoral.18749

**Published:** 2013-10-13

**Authors:** Konstanze Scheller, Alexander W. Eckert, Christian Scheller

**Affiliations:** 1 MD, DMD, Department of Oral and Maxillofacial Surgery and Facial Plastic Surgery, Martin-Luther-University Halle-Wittenberg (Head: Prof. Dr. Dr. J. Schubert), Germany; 2 MD, DMD, PhD, Associate Professor, Department of Oral and Maxillofacial Surgery and Facial Plastic Surgery, Martin-Luther-University Halle-Wittenberg (Head: Prof. Dr. Dr. J. Schubert), Germany; 3 MD, Department of Neurosurgery, Martin-Luther-University Halle-Wittenberg (Head: Prof. Dr. C. Strauss), Germany

## Abstract

Objectives: Eagle’s syndrome is caused by an elongated or mineralised styloid process and characterised by facial and pharyngeal pain, odynophagia and dysphagia. Diagnosis is based on clinical findings. However radiologic imaging, like panoramic radiograph, helps to confirm the diagnosis. There are different treatments of the Eagle’s syndrome. Anti-inflammatory medication (carbamazepime, corticosteroids) and/or surgical interventions are established. The aim of the different surgical techniques is to resect the elongated styloid process near the skull base. Study Design: A transoral, retromolar, para-tonsillar approach was performed to expose and resect the elongated calcified styloid process in a consecutive series of six patients. The use of different angled ring curettes, generally used in hypophysis surgery, facilitated the preparation of the styloid process through the surrounding tissue to the skull base, without a compromise to the surrounding tissue. Clinical examinations were performed pre- and postoperatively (3 month and after 1 year after surgery) in all patients. Results: No intra- or postoperative complications were observed. The hypophysis ring curettes facilitated the preparation of the styloid process to the skull base. Conclusions: The transoral, retromolar, para-tonsillar approach is a secure and fast method to resect an elongated symptomatic styloid process. Side effects of the classical transoral trans-tonsillar approach did not occur.

** Key words:**Retromolar, para-tonsillar approach, Eagle syndrome, clinical features.

## Introduction

Eagle’s syndrome was first described in 1937 by Eagle and refers to a rare constellation of neuropathic and vascular occlusive symptoms caused by a pathologic elongation of the styloid process and/or styloid chain (1). Eagle described two different symp-toms of the styloid syndrome (2,3). The classical Eagle’s syndrome appears after tonsillectomy or cervical trauma while the sty lo-carotid syndrome is suggested to be caused by a mechanical irritation of the sympathetic plexus in the wall of the carotid arteries (3,4).

Eagle and others stated that a normal styloid process is about 25-29 mm in length and any length beyond is elongated (1,5). Mineralisation or calcification of the styloid complex can cause this elongated styloid process and is seen in 2-28% of the general population (6). Therefore only 4-10% of all subjects with an elongated styloid process are symptomatic (7,8).

Patients with the classical Eagle’s syndrome often are misdiagnosed for a long time and treated in terms of some functional temporomandibular joint dysfunctions and disorders (TMJ) or unspecific glossopharyngeal, occipital or sphenopalatine neuralgia (8). Given to the many variations of clinical presentation of the classical Eagle’s syndrome a careful recording of the patient’s history and the present symptoms is necessary. The clinical examination and the palpation of the elongated styloid process through the fossa tonsillaris is very helpful and specific (3,8). While the styloid process normally cannot be palpated in this side, it is easy to palpate an elongated. This palpation often recreates the specific, particular neuralgia and allows a differentiation to temporomandibular joint dysfunctions and disorders (8-10). A particular face and neck pain can be specifically exacerbated by a forced neck flexion, extension and contralateral rotation in patients with Eagle’s syndrome and not in patients with TMJ (11). Other clinical symptoms like a pharyngeal foreign body sensation or dysphagia are even leading to the diagnosis of an Eagle’s syndrome (10,11).

**Figure 1:**
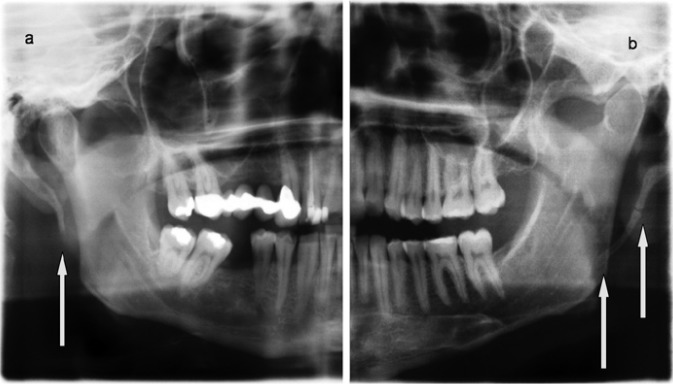
Conventional panoramic view of two patients with an Eagle’s syndrome. The calcified, elongated styloid process was elongated (a) or pseudo-articulated (b).

After an exact clinical examination a conventional radiograph (e.g. panoramic radiograph) can help to confirm the clinical diagnosis by the presentation of an elongated, calcified styloid process or styloid ligament. The elongated styloid process often presents as elongated, pseudo-articulated or segmented (12-14), (Fig. 1). A spiral CT with 3D-reconstruction can illustrate the anatomical structures in detail (11).

Amongst others the treatment depends also on the specialist who first saw the patient. The non-invasive management is the first line for the neuropathic sequelae of the Eagle’s syndrome (15). So many patients with a unspecific neuralgia caused by Eagle’s syndrome are treated pharmacologically by the application of non-steroidal anti-inflammatory medications or even carbamazepime, valporate, gabapentin or amitriptyline (2,6,9). Other pharmaceutical treatment like the transpharyngeal, image guided steroid and lidocain injection has shown no long-term substantial effectiveness and the disease recurred to the most patients after 6-12 month (10,16). The non-invasive, pharmaceutical therapy can provide temporary relief and may be an option for patients refusing surgical treatment.

The second and permanent line of the specific therapy of the Eagle’s syndrome is the surgical (9,16). The surgical shortening and resection of the abnormal styloid process, first described by Eagle (3), can lead to a persistent absence of the clinical symptoms. Two major procedures of the surgical treatment of Eagle’s syndrome are known and controversially discussed: the extraoral cervical or retroauricular approach and the classical transoral approach through the fossa tonsillaris (15,17-20).

Both have some considerable advantages and disadvantages and should be performed individually in respect to the patient’s circumstances and the surgeon’s experience.

## Material and Methods

Six patients suffering from Eagle’s syndrome were retrospectively analysed.

The surgical procedure to resect the elongated symptomatic styloid process was performed by a transoral, retromolar, para-tonsillar approach between 01/2008 – 1/2009 in our department.

-Clinical evaluation

Most patients’ complained about an ipsilateral pharyngeal foreign body sensation, a dysphagia and a painful limitation of neck mobility to the affected side ( Table 1). Other symptoms like odynophagia, painful trismus, vertigo were less specific and less common.

**Table 1:**
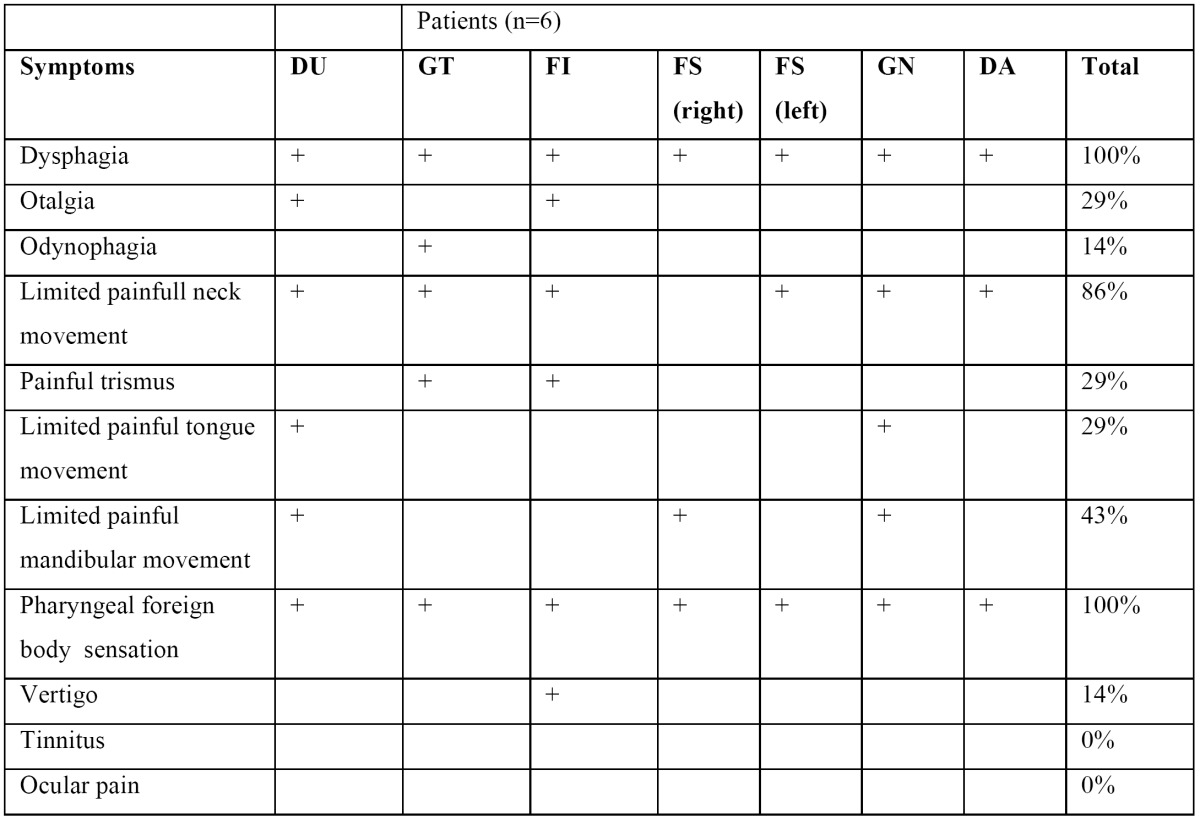
Chief preoperative (subjective) symptoms (n=7).

-Radiographic imaging

In all patients the length of the symptomatic and non-symptomatic styloid process was analysed by a digital, conventional panoramic radiograph (Orthophos Plus DS, Sirona, Wels, Austria).

The elongated and the styloid processes on the affected and non-affected side were measured (in mm) using the measurement program of the Orthophos Plus DS (by Sirona, Wels, Austria). The magnification factor (1.2-times) of the dental panoramic view was automatically corrected by using the special software SIDEXIS.

-Surgical procedure

Surgical procedures were performed in general anaesthesia. A preoperative, prophylactic single-shot antibiotic therapy was done with cefuroxime 1500 mg (INNO PHARM, 31028 Gronau/Leine, Germany).

A transoral, retromolar, para-tonsillar approach, similar to the procedure described in 2011 by Raychowdhury (21), was performed in all patients.

**Figure 2:**
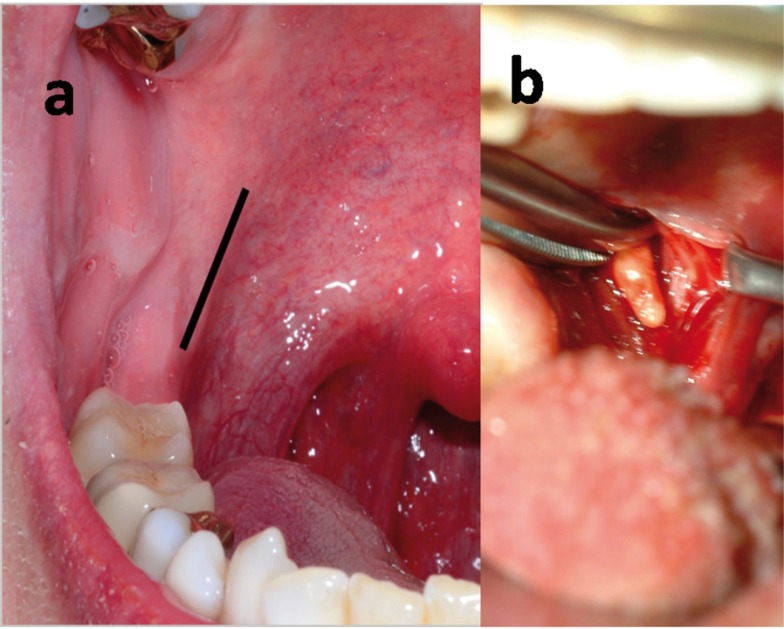
Schematic presentation of the retromolar, paratonsillar approach (a) and the tip of the elongated styloid process (b). After blunt dissection the styloid process could be identified and easily preparated to the skull base by using a round hypophysis ring curette. After the resection near the scull base by a small luer bone rongeur or neurosurgical bone punch the mucosal defect was closed primarily.

**Figure 3:**
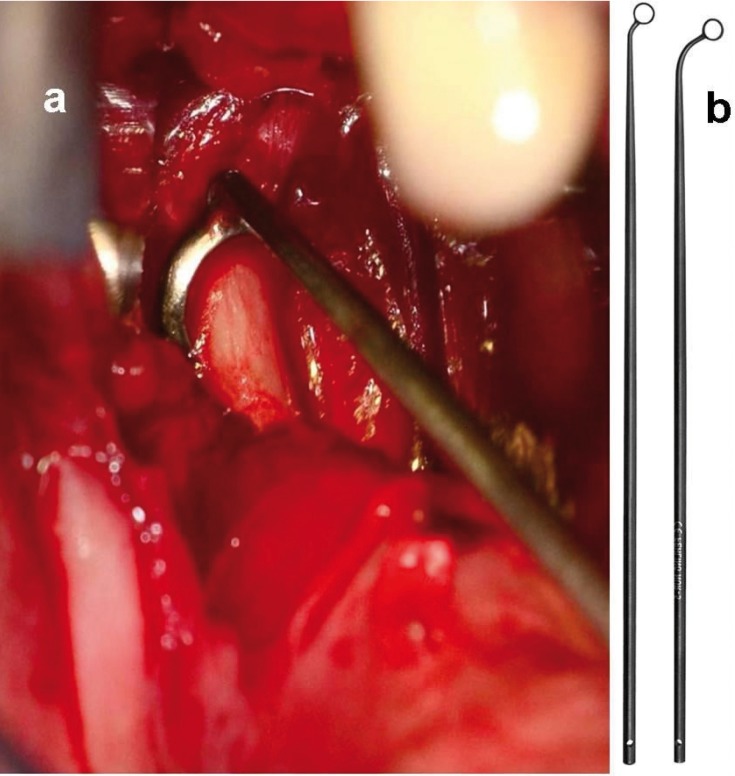
Clinical presentation of the intraoperative use of a ring curette (a). Different angled (45 to 60 degree) hypophysis ring curettes were used to prepare the styloid process to the skull base (Fehling Instruments GmbH & Co. KG, Karlstein, Germany, b).

A vertical incision of 2-3 cm was made in the lingual mucosa of the ascending upper jaw (schematic presentation, Fig. 2). The tip of the elongated styloid process was identified by deep digital palpation and exposed by blunt dissection (Fig. 2). The periost was incised on the tip of the elongated styloid process. The styloid process was stripped free of the surrounding tissue and attached ligaments by using different angled (45 to 60 degree) hypophysis ring curettes (Fig. 3) from 0.2 to 0.4 cm in diameter (Fehling Instruments GmbH & Co. KG, Karlstein, Germany). The styloid process could be easy exposed until the basis near the skull base. The free and naked styloid process was finally removed from the temporal bone near the skull base by using a small Luer Bone Rongeur or a neurosurgical bone punch (Martin GmbH & CO.KG, Tuttlingen, Germany). After removing the elongated styloid process the mucosa was primarily closed with absorbable sutures.

The patient with the bilateral symptomatic styloid process was treated separately for each side to avoid great postoperative discomfort (19).

Clinical examination was done 1 and 3 weeks, 3 month and one year postoperatively. One year after the operation an evaluation of the Eagle’s specific symptoms was done.

-Statistics

Mean values are given with standard deviations.

## Results

Six patients with the clinical and radiographic diagnosis of an Eagle’s syndrome were included in this study ( Table 2). There were two men and four women. One woman showed a bilaterally symptomatic styloid process and was treated separately for each side (n=7).

**Table 2:**
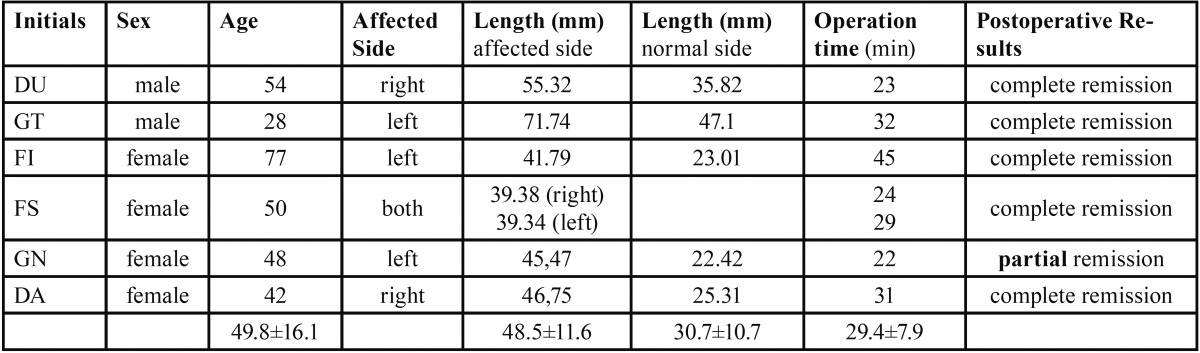
Distribution of patients, gender, age, individual length of the symptomatic and asymptomatic styloid process, operation time and the postoperative outcome.

The patients’ mean age was 49.8±16.1 years (range from 28 to 77 years). None of the patients had a previous history of tonsillectomy or head and neck trauma.

Radiographic imaging showed different forms of the elongated, symptomatic styloid processes on the affected side, like elongated (Fig. 1) or pseudo-articulated (Fig. 1). The elongated styloid process (conventional panoramic radiograph) was 48.5±11.6 mm on the affected and 30.7±10.7 mm on the non affected side ( Table 2). Compared to the findings of Eagle the styloid process (normally: 25-29 mm) was even elongated on the asymptomatic side in these patients (1,5). So it seems that these patients tended to develop an elongation (22).

Postoperative all patients experienced moderate pain in the first week and mild dysphagia for 2 to 3 weeks. One year after the operation an evaluation of the Eagle’s specific symptoms was done. A complete remission of all preoperatively stated complaints and symptoms was seen in 5 patients. Only one patient (female, 48 years) still complained about a less extensive pharyngeal foreign body sensation (partial remission,  Table 2).

The surgical procedure was easy to perform and fast (operation time: 29.4±7.9 min). No intra- or postoperative operation-associated side effects like retropharyngeal infection or airway edema were seen ( Table 3). There was a moderate swelling of the mucosa for 3-4 days. Wound healing was in time and there was no postoperative infection.

**Table 3:**
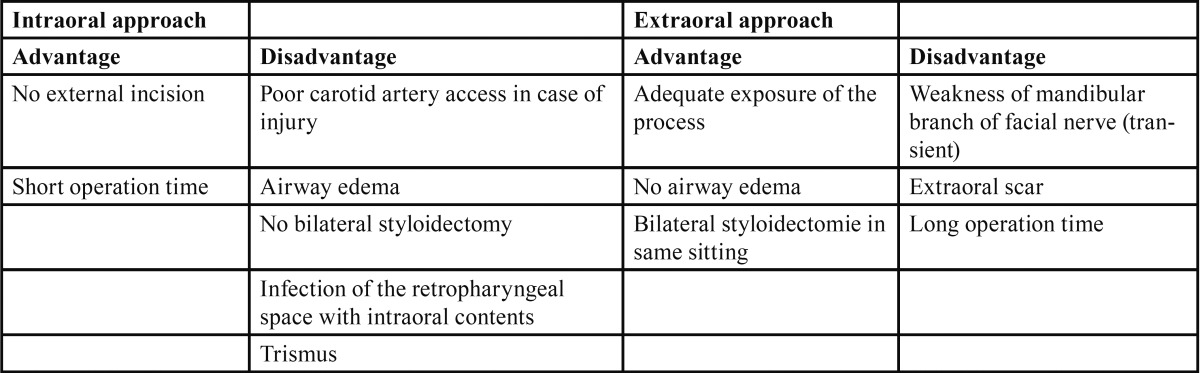
Advantages and disadvantages of the intra- and extroral approach to resect the styloid process.

## Discussion

The elongation of the styloid process is not uncommon, but the true Eagle’s syndrome is a rare disease (6,10). Most patients with an elongated and mineralized styloid process are asymptomatic and do not need any treatment. When symptoms exist the severity of the symptoms does not correlate with the length or the extent of the mineralized process (8). So the diagnosis of an Eagle’s syndrome is often difficult and the differential diagnoses include all conditions causing orofacial pain (10,15,23). Medical history is the main guide to diagnosis, however palpation of the elongated styloid process and radiological examination (panoramic radiograph, computed tomography) are combined to confirm the diagnosis (11). The conventional panoramic radiograph is the preferred radiological examination in our department, because the elongation of the styloid process can be demonstrated clearly and the effective radiation dose (0.002 mSv) is low (24). A spiral CT with 3D-reconstruction (bone window) can illustrate the anatomical structures of the styloid process more in detail (11), but the effective radiation dose (about 1.89 – 0.2 mSv) is 100-1000-times higher than a conventional orthopantomogramm. So the choice of the radiologic imaging has to be considered carefully according to the International Atomic Energy Agency radiological protection of patients.

Eagle’ syndrome can be treated conservatively, surgical or both. Nonsurgical treatment with steroid hormones, anti-epileptic and antihistamine drugs were often proposed for the treatment of Eagle’s syndrome. Clinical studies showed, that there is no real long term positive effect and 6-12 month after the conservative, non-surgical treatment the symptoms often recurred (9,16). The most satisfying and effective method to eliminate the symptoms caused by an elongated styloid process is its surgical shortening (3,22).

Different surgical procedures and approaches to the styloid process have been described in literature so far (17-23). All of them seem to have some considerable advantages and disadvantages (Table 3). The external approach is favoured by some surgeon because of its adequate exposure of the process and the associated structures, the transoral approach because of its simplicity and velocity (11,22). However there are severe complications that have to be considered. The external incision and postoperative scar on the neck can be avoided by using a transoral approach, but the poor visualisation of the operation field is a considerable disadvantage. Matsumoto et al. described the benefit to identify small vessels and nerves and allows the surgeon to avoid injuries by the use of an endoscope at the beginning of the operation (25). In case of an intra-operative injury of the carotid arteries with massive bleeding there is only a poor access to the anatomical structures. The exposure of the retropharyngeal space to the intraoral contents may even elevate the infection risk by intraoral bacterial (23). The transoral, retromolar, para-tonsillar approach performed in this study showed no severe side effects (21). There were no intraoperative complications like bleeding or nerve injury and no postoperative infection or airway oedema.

In conclusion, the intraoral, retromolar, para-tonsillar approach is a good method to treat patient with a clinically and radiological approved Eagle’s syndrome. The use of a hypophysis ring curette facilitates the preparation of the styloid process to the skull base without any damage of the surrounding tissue. The resection can be easily done with a small luer bone rongeur or a neurosurgical bone punch.

The poor visualisation of the operation field in a transoral approach needs the surgeon to be experienced and to be familiar with the anatomical structures, the operation technique and the handling of possible complications. An endoscopic resection control on the skull base can be useful.
